# A Triplet Label Extends Two‐Dimensional Infrared Spectroscopy from Pico‐ to Microseconds

**DOI:** 10.1002/anie.202211490

**Published:** 2022-11-09

**Authors:** Hendrik Brunst, Hafiz M. A. Masood, A. Rafael Thun, Alexander Kondratiev, Georg Wille, Luuk J. G. W. van Wilderen, Jens Bredenbeck

**Affiliations:** ^1^ Institut für Biophysik Goethe Universität Frankfurt Max-von-Laue-Straße 1 60438 Frankfurt Germany

**Keywords:** 2D-IR, IR Spectroscopy, Laser Spectroscopy, Spectral Diffusion, VIPER

## Abstract

In conventional two‐dimensional infrared (2D‐IR) spectroscopy, the inherently short vibrational lifetimes limit the time window to observe molecular dynamics typically to tens of picoseconds. The rather complex dynamics of organized molecular systems (e.g., glass formers, polymers, membranes, proteins), however, span a wide range of timescales from femto‐ to microseconds and beyond. Vibrationally Promoted Electronic Resonance (VIPER) 2D‐IR negates the limitations of 2D‐IR spectroscopy, for its signal decays with the electronic lifetime. Here, we present 2‐Isopropylthioxanthone as the first VIPER 2D‐IR probe to exploit intersystem crossing, thereby covering even the microsecond timescale. We achieved the required signal‐to‐noise ratio and resolution by introducing the Fourier‐transform approach to the VIPER 2D‐IR pulse sequence. Now, we are in a position to monitor dynamics via spectral diffusion several orders of magnitude beyond the vibrational lifetime of 2D‐IR labels.

## Graphical abstract

Short vibrational lifetimes limit monitoring molecular dynamics by conventional IR spectroscopy to ≈15 ps. We extend this window ≈50 000 fold by introducing the novel Fourier‐transform VIPER pulse sequence with the triplet label 2‐Isopropylthioxanthone. Slow processes up to 1 μs (e.g., in glassy matrices, liquid crystals, or solvent dynamics) are now accessible.

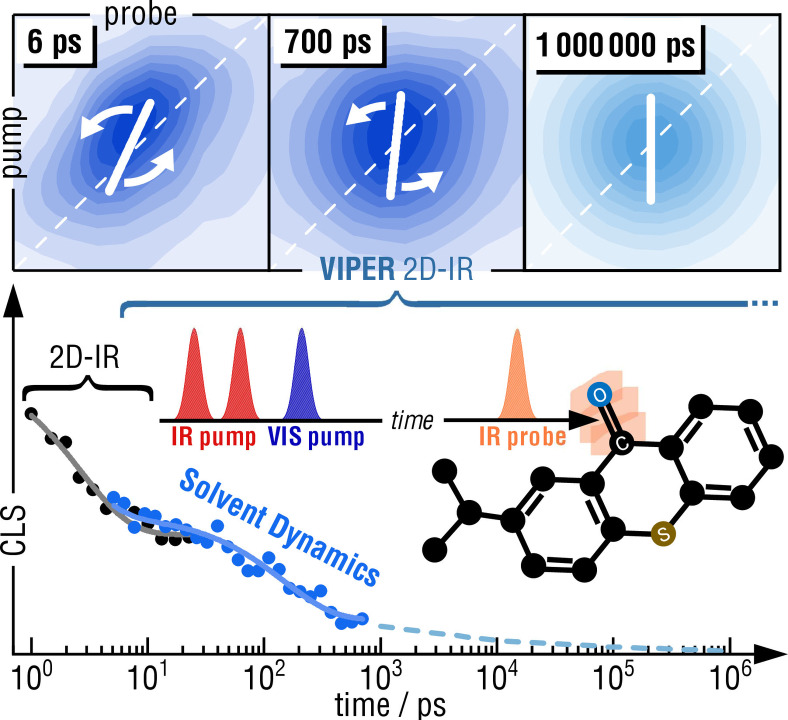

Two‐dimensional infrared spectroscopy (2D‐IR) is a well‐established method for investigating ultrafast equilibrium processes on molecular scales. It has been successfully employed to resolve dynamics of solvation,^[1]^ membrane water interactions,^[2]^ protein dynamics,^[3]^ liquid‐liquid interfaces,^[4]^ crowding,^[5]^ nanoconfinement,^[6]^ and liquid crystals^[7]^ to name a few. 2D‐IR detects dynamics via the frequencies of vibrations that are modulated by structure changes in their surroundings, a process called spectral diffusion.^[8]^ The frequency fluctuations report e.g. about changes in local electric fields, H‐bonding and van der Waals interactions.^[9]^ However, the short lifetimes of vibrations limit the time scale of dynamics detectable by 2D‐IR, typically to the lower picosecond range.[^2^, ^10^] Liquids consisting of small molecules, such as water, sample all possible molecular configurations within a few picoseconds, as reported by spectral diffusion which is completed on this time scale.[^11^, ^12^] Yet, dynamics of organized molecular systems as the ones mentioned above can span a wide continuum of timescales. An example is provided by proteins, where dynamics extends from fluctuations of dihedral backbone angles on the sub‐picosecond time scale,^[13]^ to domain motions and folding and unfolding processes into the millisecond range. Similarly, in membranes dynamics is extending from the picosecond dynamics that modulate membrane water interaction^[14]^ or generate large electric field fluctuations,^[2]^ to much slower diffusive processes, such as e.g. the exchange of ions at the membrane or the formation of lipid‐protein complexes. Also, in systems with less complex constituents, such as glass‐forming liquids^[15]^ or liquid crystals^[16]^ dynamics can extend over a wide range of time scales. Hence, while 2D‐IR provides important insight in ultrafast processes, only a fraction of the actual dynamics of complex molecular systems is accessible for conventional 2D‐IR spectroscopy,[^11^, ^14^, ^17^] which is relying on vibrational marker bands or specifically introduced IR labels with typical vibrational lifetimes in the low picosecond range.

To break the shackles of conventional 2D‐IR spectroscopy, we have developed the VIbrationally Promoted Electronic Resonance (VIPER) 2D‐IR pulse sequence.^[18]^ The first version of the VIPER pump pulse sequence was composed of one narrowband IR pump pulse, followed by an off‐resonant visible (VIS) pulse. The vibrational excitation red‐shifts the VIS spectrum of the molecules into resonance with the formerly off‐resonant VIS pump pulse. Thereby, VIPER transfers only the vibrationally excited population to an electronically excited state (Scheme 1c). This prevents the decay of the signal caused by vibrational relaxation. Now, the 2D‐IR bleach signal lives as long as the electronically excited state. At the same time, it allows one to obtain the vibrational frequency fluctuations of the IR transition in the electronic ground state of the molecule (S_0_), which contains the desired information on dynamics. Note that the VIPER 2D‐IR pulse sequence is different from the triggered exchange 2D‐IR sequence, which uses a resonant instead of an off‐resonant VIS pulse and whose signal decays with the vibrational life time.^[19]^

**Scheme 1:**
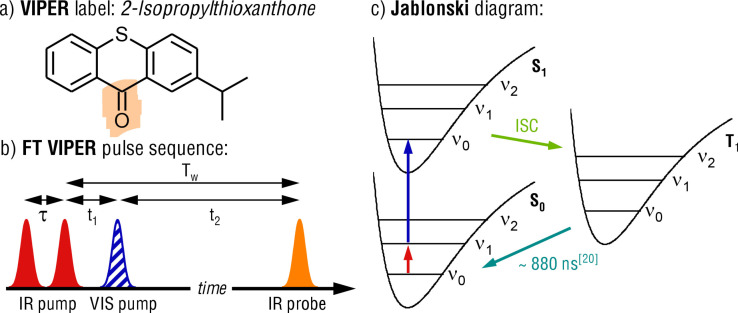
a) Chemical structure of the VIPER label 2‐ITX. The used marker moiety C=O is highlighted. b) Pulse sequence of the VIPER FT 2D‐IR experiment. The coherence time τ is the delay between the IR pump pulses. t_1_ and t_2_ are the delays between IR_pump_↔VIS_pump_ and VIS_pump_↔IR_probe_ pulses, respectively. The VIS pulse is applied during the population time T_w_ between the IR pump and probe pulses. The VIS pump pulse is striped to indicate its off‐resonant nature. c) Jablonski diagram of the VIPER excitation. The red arrow corresponds to the IR pump excitation. The vertical blue arrow corresponds to the VIS excitation. The green arrow represents the ISC from the S_1_ to the T_1_ state. The cyan arrow depicts the relaxation to the ground state.

Here we introduce a Fourier‐transform (FT) implementation of VIPER, which substantially increases the achievable signal‐to‐noise ratio and resolution of VIPER 2D‐IR and allows routine monitoring of 2D‐IR label dynamics far beyond their inherent vibrational lifetime. We furthermore introduce the 2‐Isopropylthioxanthone (2‐ITX) moiety as the first VIPER probe or label. We will show that 2‐ITX, which efficiently crosses from a singlet to a triplet state (intersystem crossing—ISC) upon VIS excitation (quantum yield ≈0.86), extends the accessible time window for 2D‐IR experiments in principle to microseconds with its triplet lifetime of 880 ns.^[20]^ As an exemplary case for the significant extension of the 2D‐IR time window, we monitor spectral diffusion of 2‐ITX′s C=O oscillator in the viscous environment of an ionic liquid.

First, we will recapitulate the information content in conventional 2D‐IR and exemplify its limitations on our label 2‐ITX in a model system, an ionic liquid, that features long lasting spectral diffusion dynamics. Next, we will explain the concept of VIPER 2D‐IR spectral diffusion measurements and how it overcomes these limitations. We will present the new VIPER FT 2D‐IR approach and the first triplet VIPER label 2‐ITX. Finally, we will show, how the combination of these enables us to measure spectral diffusion on formerly inaccessible timescales up to microseconds.

The vibrational frequency of an IR oscillator reports on interactions with its environment because those deform the potential of the oscillator, thereby shifting its vibrational frequency. The different environments lead to a distribution of oscillator frequencies. Therefore, 2D‐IR can sample the environments of these oscillators as a function of time. This is done by selective excitation of a sub‐ensemble of the frequency distribution, and subsequent measurement of its frequency after a variable waiting time T_w_. The frequency of the selective excitation ω_pump_ is then plotted versus the frequency of the probe beam ω_probe_ for every T_w_. Selective excitation can be achieved either by a narrowband IR pump pulse tuned across the frequency range of interest or by exciting with a broad band pulse pair the delay of which is scanned in the time domain and subsequently Fourier‐transformed (FT).^[21]^

FT 2D‐IR spectra at selected delays of 2‐ITX in an ionic liquid (1‐Hexyl‐3‐methylimidazolium bis(trifluoromethylsulfonyl)imide, HMIM NTf_2_) are shown in Figure 1a–d. Panel a shows the bleach of the C=O marker band that is elongated along the diagonal after 1 ps. The C=O band is actually composed of two separate bands, attributed to a Fermi resonance (see Figure S1).^[22]^ The low frequency part is used for the subsequent analysis. The viscous ionic liquid was chosen, as a prime example for a slowly changing environment. At short delay times of 1 ps the environment has barely changed, resulting in a strong correlation between ω_pump_ and ω_probe_, which is visible from the diagonally elongated 2D‐IR band shape. With increasing delay time, the local fluctuations of the environment reduce the correlation, this effect is called spectral diffusion. This randomization of the environment is reflected by the more upright shape of the signal at longer T_w_ (compare panels b–d). When the correlation is completely lost, the line shape is circular, i.e., each cut along the probe axis will look identical except for the amplitude. The spectral diffusion can be extracted from the time evolution of the 2D‐IR line shapes, for instance via center line slope (CLS) analysis.^[23]^ Panel e shows the CLS analysis of the low frequency peak of the C=O signature. A monoexponential fit to the time evolution of the CLS yields a 2 ps lifetime with an amplitude of 0.46, but an offset of 0.37 remains, for all delays at which the bleach signal has not fully decayed due to the vibrational lifetime. At longer delays the CLS cannot be measured as the bleach has disappeared. This significant CLS offset is the result of a not yet randomized environment within the vibrational lifetime‐limited time window of conventional 2D‐IR. Evidently, after even ≈25 ps the environment is still rather correlated to its initial structure.

**Figure 1:**
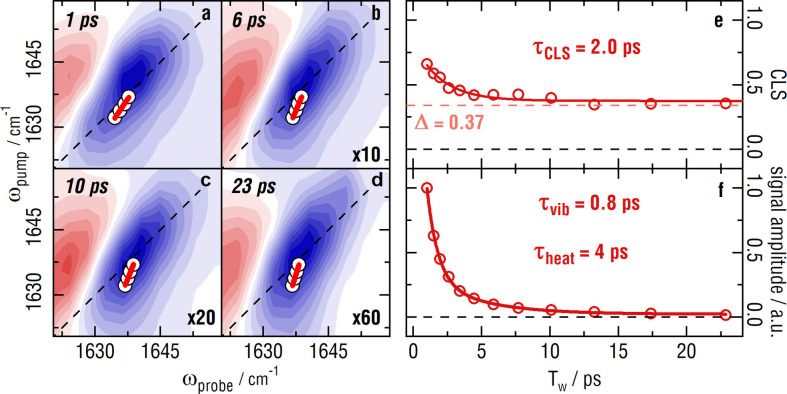
2D‐IR data of 2‐ITX in HMIM NTf_2_. a–d) C=O bleach of 2‐ITX at Tw=1 ps, 6 ps, 10 ps and 23 ps, respectively. The spectra use the same color coding and are scaled by the factors given in the bottom right to match the amplitudes. The white dots underlying the red line are the maxima of the gaussian fits to cuts along the probe axis. Fits were applied from 1632 cm^−1^ to 1637 cm^−1^. The red line itself is the linear fit to said maxima. The inverse of the slope of that fit is the CLS. e) illustrates the CLS decay with T_w_. A monoexponential fit and the according lifetime τ_CLS_ and offset Δ are shown as well. f) shows the normalized integrated signal size of a 2D‐Gaussian fit to the same spectral region as used for the CLS analysis. A biexponential fit and its attributed (vibrational and heating) lifetimes are stated as well.

The decrease in integrated signal size with T_w_ of the same spectral region is shown in panel f. The signal decay follows a bi‐exponential behavior, yielding 0.8 ps and 4 ps lifetimes with amplitudes of 2.4 and 0.4 respectively. The fast 0.8 ps component reflects vibrational relaxation, while we assign the 4 ps lifetime to the decay of a small hot ground state signal remaining after vibrational relaxation (note that Figure 1d′s amplitude is scaled by a factor 60 relative to panel a). Our system exemplifies that the time window accessible via conventional 2D‐IR spectroscopy is significantly too short to elucidate the full dynamics of complex systems, even for highly concentrated samples (i.e. generating large signals) and even if a hot ground state band is available (which sometimes is measurable before energy transfer from the molecule to the environment). This problem we overcome with the VIPER FT 2D‐IR pulse sequence.

In the VIPER pulse sequence (see Scheme 1b) the selective IR pump interactions excite a sub‐ensemble of molecules to the vibrationally excited state (ν_0_→ν_1_) (see Scheme 1c), just as in conventional 2D‐IR spectroscopy. This vibrational excitation can shift the VIS absorption spectrum to higher wavelengths in two ways: by coupling of the electronic transition either to the excited vibrational mode or to a lower energy mode populated by internal vibrational relaxation (IVR) of the initially excited mode.^[24]^ A VIS pulse that is resonant with the shifted VIS transition can then transfer these pre‐excited molecules to an electronically excited state (S_1_). The selectivity of vibrational excitation by IR is thus combined with an electronic excitation, which can populate states that have a substantially longer lifetime than vibrational excitations. VIPER with a narrowband IR excitation has already been used to measure chemical exchange based on singlet electronic excitation^[18]^ as well as to control photochemical reactions in mixtures.^[25]^ Both of these applications used the IR narrow band excitation to distinguish between separate bands. Here, we distinguish sub‐populations *within* one inhomogeneously broadened band, that interconvert as reflected by spectral diffusion. We demonstrate extension of the observation time window to the much longer triplet time scales. To put this into practice, we introduce the first triplet VIPER label, as well as the VIPER FT 2D‐IR implementation, which yields significantly higher resolution and data quality.

We replaced the previously used narrowband IR pump pulse by a pulse pair with a variable delay τ, generated by an interferometer. This approach increases the time resolution of the experiment significantly (down to the 100 fs‐pulse duration; Fabry‐Perot generated narrowband pulses are typically 1–1.5 ps long). The FT of the time‐domain signal as a function of τ yields the 2D‐IR spectrum. Simultaneously, the VIS pump beam is mechanically chopped at half the repetition rate of the laser. In this fashion, a conventional 2D‐IR spectrum as well as a 2D‐IR spectrum with VIS excitation is measured quasi‐simultaneously. The difference of these spectra yields the VIPER FT 2D‐IR spectrum.

In the VIPER pulse sequence, the delay *t_1_* (see Scheme 1) is set to a value that prevents coherent artifacts caused by interactions of the IR and VIS pump beams. With the FT approach *t_1_* can be shorter, yielding larger signals because less population decay from the ν_1_ state occurs before excitation to the S_1_ state. Implementing the FT approach significantly enhanced our signal‐to‐noise ratio. Now, the routine use of labels even with low extinction coefficients is in reach with VIPER 2D‐IR spectroscopy. In contrast to conventional 2D‐IR experiments, the data analysis is even simplified for VIPER, because the bleach shape is not distorted by an excited state absorption (ESA) due to its signal being “removed” by the electronic excitation (see Scheme 1c).

Figure 2 shows the VIPER FT 2D‐IR spectra of 2‐ITX in HMIM NTf_2_. Panel a shows the spectrum of the C=O at 1637 cm^−1^ (*diagonal peak 2*) and of two overlapping ring modes around 1593 cm^−1^ (*diagonal peak 3*) at T_w_=700 ps. Please note, that *peak 1* and *4* are *not* conventional 2D‐IR cross peaks caused by anharmonic coupling or chemical exchange, but inherent features of VIPER 2D‐IR spectroscopy. We assign the VIPER 2D‐IR spectrum as follows: The pre‐excitation of the C=O (*peak 2*) enables absorption of VIS photons and, thereby, promotion of the molecule to the S_1_ state. This generates the *cross peak 1*, because the whole molecule is now in a different electronic state. Evidently, in VIPER spectroscopy, anharmonic couplings and/or chemical exchange are therefore *not* a prerequisite for a cross peak between vibrations. Yet, vibrations need to belong to the same molecule and be changed by the electronic excitation. In fact, potential correlation between the environments of two oscillators could be analyzed via the VIPER approach. *Peak 1* and *2* are considerably larger than *peak 3* and *4*, because in 2‐ITX the C=O mode displays a stronger coupling to the electronic transition than its ring modes.^[24]^

**Figure 2:**
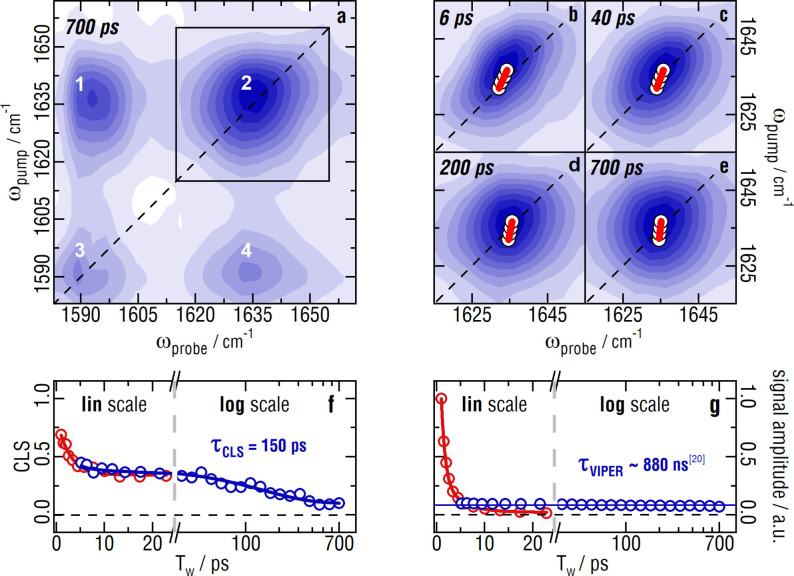
VIPER FT 2D‐IR data of an identical 2‐ITX sample as shown in Figure 1. a) shows the VIPER 2D‐IR spectrum at T_w_=700 ps in a broader spectral range. The FTIR absorption spectrum is plotted on the top and right for comparison. The black square indicates the spectral range plotted in b–e. b–e) illustrate peak 2 at T_w_=6 ps, 40 ps, 200 ps and 700 ps, respectively. The white dots underlying the red line are the maxima of the gaussian fits to cuts along the probe axis. The superimposed red line symbolizes the linear fit to said maxima. The inverse of the slope of that fit is the CLS. f) illustrates the CLS decay with T_w_, compared to the CLS decay measured via conventional 2D‐IR. g) shows the normalized integrated signal size in comparison to the conventional 2D‐IR signal size.

Panels b‐e show the C=O bleach *peak 2* at 6 ps, 40 ps, 200 ps and 700 ps, respectively. The spectral diffusion is clearly visible from the bleach turning upright. Panel f shows the CLS analysis of *peak 2* (in blue) in comparison to the results from conventional 2D‐IR under identical sample conditions (in red; the same analysis is presented in Figure 1e). The CLS curves are virtually identical, for as long as the conventional 2D‐IR signal is measurable (up to ≈25 ps). In principle it is possible, that the CLS dynamics of 2D‐IR and VIPER 2D‐IR differ: 2D‐IR shows a combination of dynamics in the vibrational ground and the vibrationally excited state, and there are cases where vibrational excitation alters the dynamics of the system.^[26]^ VIPER 2D‐IR eliminates the contribution from the vibrationally excited state and can yield unperturbed equilibrium dynamics. For longer times, the VIPER FT 2D‐IR now reveals a second time constant of 150 ps, which is inaccessible for conventional 2D‐IR spectroscopy and therefore results in an offset of about 0.3 in the CLS curve in the conventional experiment.

The VIPER signal is analyzed for T_w_ >6 ps, for it overlaps with a triggered exchange signal before.[^18^, ^19^] The latter signal decays with the lifetimes of the conventional 2D‐IR signal of the C=O (0.8 ps, 4 ps; see Figure 1f) and is therefore negligible from 6 ps onwards. The evolution of the CLS can consequently be extracted from conventional 2D‐IR spectra for early delays (≈2–3× vibrational lifetime), and via VIPER 2D‐IR once the conventional 2D‐IR signal disappears due to vibrational relaxation. The triplet lifetime of 2‐ITX is 880 ns,^[20]^ in principle allowing to access even these slow timescales with VIPER FT 2D‐IR. Spectral diffusion can thus be measured from essentially no delay to a few microseconds for our label. By applying synchronized lasers we will be able to overcome the limitation of mechanical delay stages, which currently restricts our measurements to 700 ps.^[27]^ Panel g shows the comparison of the (integrated) signal sizes of conventional 2D‐IR and VIPER FT 2D‐IR. The signal sizes are similar at about 6 ps. The conventional 2D‐IR signal size is however larger at shorter T_w_, because it is a third‐order spectroscopy, compared to VIPER 2D‐IR which is a fifth‐order spectroscopy. Even though the VIPER 2D‐IR signal starts out smaller, it quickly outperforms the conventional 2D‐IR due to its significantly longer lifetime (>5 orders of magnitude longer).

In conclusion, we have introduced VIPER FT 2D‐IR, thereby greatly improving the sensitivity and resolution of the VIPER 2D‐IR approach. We have established 2‐ITX as the first VIPER label for spectral diffusion measurements. The combination of the two enabled us to measure the spectral diffusion exemplary in the viscous ionic liquid HMIM NTf_2_, where both fast and slow molecular motions are present. Accessibility of the longer time scale is granted by the long triplet lifetime of the new VIPER label in form of the 2‐ITX moiety, which could be used as a dissolved probe molecule, but also turned into a VIPER label that can be covalently attached to the system of interest, e.g. in form of a noncanonical amino acid.^[28]^ Even though the measurements here used a high concentration of 200 mM, this value can be easily reduced below 20 mM, judged from the available data quality. This value can be further reduced to the single digit mM range with straightforward modifications of the setup to increase the IR intensity, which is the range where e.g. conventional 2D‐IR spectroscopy on proteins is operating.^[11]^ Future design efforts for VIPER labels will take into account and optimize the vibronic couplings responsible for the VIPER signal size.^[24]^ To exploit the entire lifetime of the 2‐ITX VIPER 2D‐IR signal electronically synchronized lasers are necessary, as T_w_ is limited by the length of the mechanical delay stage otherwise. Even significantly slower dynamics up to the microsecond timescale can then be investigated with 2‐ITX and VIPER FT 2D‐IR, thereby granting new insights into systems and phenomena displaying a wide range of time scales, such as proteins, glass formers, liquid crystals or membrane systems.

## Conflict of interest

The authors declare no conflict of interest.

## Supporting information

As a service to our authors and readers, this journal provides supporting information supplied by the authors. Such materials are peer reviewed and may be re‐organized for online delivery, but are not copy‐edited or typeset. Technical support issues arising from supporting information (other than missing files) should be addressed to the authors.

Supporting Information

## Data Availability

The data that support the findings of this study are available from the corresponding author upon reasonable request.
