# Supplementary material for: A Triplet Label Extends Two‐Dimensional Infrared Spectroscopy from Pico‐ to Microseconds
Source: Angew Chem Int Ed Engl. 2022 Nov 9;61(49):e202211490. doi: 10.1002/anie.202211490 (PMC10098663; doi:10.1002/anie.202211490)
Supplement: Supplementary file 1 — Supporting Information [file ANIE-61-0-s001.pdf]

Angewandte Chemie
Eine Zeitschrift der Gesellschaft Deutscher Chemiker

Supporting Information

# A Triplet Label Extends Two-Dimensional Infrared Spectroscopy from Pico- to Microseconds

*H. Brunst, H. M. A. Masood, A. R. Thun, A. Kondratiev, G. Wille, L. J. G. W. van Wilderen, J. Bredenbeck**

## Table of Contents

**Experimental Procedures** .............................................................. **2**
Sample preparation and FTIR measurements ......................... 2
Ultrafast measurements.......................................................... 2
**Results and Discussion** ................................................................ **2**
Fermi Resonance .................................................................... 2
**References** ...................................................................................... **3**
**Author contributions** ..................................................................... **3**

# Experimental Procedures

## Sample preparation and FTIR measurements

2-Isopropylthioxanthone (2-ITX, ≥98.0%) was purchased from Tokyo Chemical Industry (TCI, Japan) and used without further purification. It was dissolved in 1-Hexyl-3-methylimidazolium bis(trifluoromethylsulfonyl)imide (HMIM NTf2, ≥99.5%), purchased from Ionic Liquid Technologies (iolitec, Germany). The ~200 mM sample was filled into a flow cell with an optical pathlength of 50 µm.[1] FTIR spectra were acquired with a Tensor 27 spectrometer (Bruker Optics) equipped with a mercury cadmium telluride (MCT) detector (InfraSpecs).

## Ultrafast measurements

The two dimensional spectra were collected using a Ti:Sa regenerative amplifier (3.5 mJ, 800 nm, 90 fs, 1 kHz, Tsunami-Spitfire XP combination of Spectra-Physics, Newport, USA) that pumps three home-built optical parametric amplifiers (OPAs).[2] The first OPA generates the IR probe pulses by difference frequency generation (DFG) of signal and idler. It was split into a probe pulse and reference pulse. The second OPA generates the IR pump pulses by difference frequency generation (DFG) of signal and idler. The third OPA generates visible pump pulses at 417 nm by sum frequency generation (SFG) between its laser fundamental and the second harmonic of its idler.

The used excitation powers were 2.3 µJ in the visible and about 3 µJ in the IR. The probe and reference pulses were dispersed using a spectrometer (Triax, Jobin Yvon) with a 150 lines/mm grating onto a 2x32 pixel MCT detector (Infrared Associates, USA). The resolution was 4 $cm^{-1}$ in the probe axis. For continuous data acquisition the detector Dewar was automatically refilled with liquid nitrogen.[3]

For time-domain 2D-IR spectroscopy, pump pulses were split into pulse pairs with adjustable delay $\tau$ using a Mach-Zehnder interferometer.[4] $\tau$ was scanned up to a coherence time of 4 ps. Fourier-transformation along $\tau$ with phase correction and a cosine function for apodization yielded the purely absorptive 2D-IR spectra. Pump scatter was suppressed using a Zinc selenide Brewster window oscillating at 250 Hz.[5] The delay of the visible pump (oriented at magic angle) and IR pump (oriented at magic angle) pulses with respect to the IR probe pulse was changed by changing the optical pathlength travelled using motorized translation stages. An optical chopper was used to collect 2D-IR signals with and without visible excitation for VIPER 2D-IR spectra. The sample was continuously pumped using a mzr-2942 pump (HNP microsystems, Germany) and moved up and down with a speed of 1 mm/s using an LTA Precision Motorized Actuator (Newport, USA) translation motor.

The 2D-IR spectra shown in Figure 1 (see main text) were averaged for 1.7 * $10^6$ shots for most delays and up to 8.4 * $10^6$ shots for the last delay, due to its smaller signal size. The VIPER FT 2D-IR spectra shown in Figure 2 (main text) were averaged for 3.1 * $10^6$ shots for all delays. 2D-data were analyzed using a home-built MatLab software.

# Results and Discussion

## Fermi Resonance

The FTIR spectrum of the laser sample of 2-ITX in HMIM $NTf_2$, displaying a Fermi-resonance of the C=O band around 1635 $cm^{-1}$. The lower frequency band at ~1634 $cm^{-1}$ was used for the center line slope analysis in Figure 1 and 2 (see main text).

**Figure S1.** (a) The FTIR spectrum of 2-ITX in HMIM $NTf_2$. (b) Second derivative of the spectrum shown in a. The dashed lines indicate the two bands of which the carbonyl band is composed. The green line indicates the band used for analysis in Figure 1 and 2 shown in the main text.

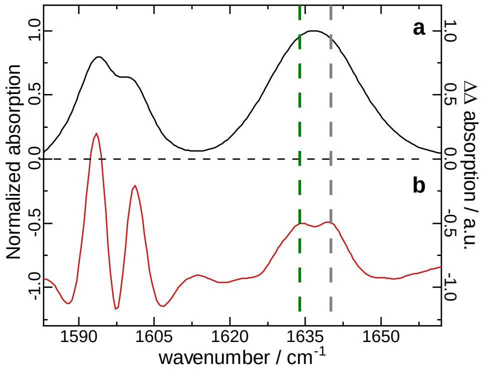

## References


[1] J. Bredenbeck, P. Hamm, *Rev. Sci. Instrum.* **2003**, *74*, 3188.
[2] P. Hamm, R. A. Kaindl, J. Stenger, *Opt Lett* **2000**, *25*, 1798.
[3] E. Deniz, K. B. Eberl, J. Bredenbeck, *Rev. Sci. Instrum.* **2018**, *89*, 116101.
[4] J. Helbing, P. Hamm, *J. Opt. Soc. Am. B* **2011**, *28*, 171.
[5] R. Bloem, S. Garrett-Roe, H. Strzalka, P. Hamm, P. Donaldson, *Opt Express* **2010**, *18*, 27067.


## Author Contributions

| | | |
|---|---|---|
| Conceptualization: | J.B., L.vW. (lead) | H.B. (supporting) |
| Data curation: | H.B. (lead) | L.vW. (supporting) |
| Formal analysis: | H.B. (lead) | |
| Funding acquisition: | J.B. (lead) | L.vW. (supporting) |
| Investigation: | H.B. (lead) | H.M. (supporting) |
| Methodology: | J.B., L.vW., H.B., A.R.T., A.K. (equal) | |
| Project administration: | J.B., L.vW., H.B. (equal) | |
| Resources: | H.B. (lead) | H.M., A.R.T., A.K. (supporting) |
| Software: | A.R.T., A.K. (lead) | G.W. (supporting) |
| Supervision: | J.B., L.vW. (lead) | G.W. (supporting) |
| Validation: | H.B. (lead) | J.B., L.vW. (supporting) |
| Visualization: | H.B. (lead) | |
| Writing - original draft: | H.B. (lead) | |
| Writing – review & editing: | J.B., L.vW. (lead) | H.M. (supporting) |
